# Sunitinib in pediatric patients with advanced gastrointestinal stromal tumor: results from a phase I/II trial

**DOI:** 10.1007/s00280-019-03814-5

**Published:** 2019-04-20

**Authors:** Arnauld C. Verschuur, Viera Bajčiová, Leo Mascarenhas, Reza Khosravan, Xun Lin, Antonella Ingrosso, Katherine A. Janeway

**Affiliations:** 1 Department of Pediatric Hematology and Oncology, Hôpital d’Enfants de la Timone, Assistance Publique-Hôpitaux de Marseille, 13005 Marseille, France; 2 University Hospital Brno–Children’s Hospital, Brno, Czech Republic; 3 Division of Hematology, Oncology, and Blood and Marrow Transplantation, Department of Pediatrics, Keck School of Medicine, Children’s Hospital Los Angeles, University of Southern California, Los Angeles, CA USA; 4 Pfizer Inc, San Diego, CA USA; 5 Pfizer S.r.L, Milan, Italy; 6 Pediatrics, Dana-Farber/Boston Children’s Cancer and Blood Disorders Center, Boston, MA USA

**Keywords:** Sunitinib, Gastrointestinal stromal tumor, Pediatric, Pharmacokinetics, Safety

## Abstract

**Background:**

Sunitinib is approved for treatment of adults with imatinib-resistant gastrointestinal stromal tumor (GIST) or imatinib intolerance.

**Methods:**

This single-arm, multicenter, multinational phase I/II clinical trial (NCT01396148) enrolled eligible patients aged 6 to < 18 years with advanced, unresectable GIST with non-mutant *KIT*, or who demonstrated disease progression or intolerance to imatinib. Patients received sunitinib 15 mg/m^2^ per day, 4-weeks-on/2-weeks-off (schedule 4/2), for ≤ 18 cycles over 24 months. Intra-patient dose escalation to 22.5 and subsequently 30 mg/m^2^ were permitted based on individual patient tolerability and supported by real-time pharmacokinetics (PK). Primary objective was PK characterization. Secondary objectives included safety, antitumor activity and PK/pharmacodynamic relationships.

**Results:**

Six patients were enrolled with median (range) age of 14 (13–16) years. All six patients completed at least three treatment cycles, with one completing all 18 cycles. Five patients had a dose increase to 22.5 mg/m^2^; two of them had a further dose increase to 30 mg/m^2^. The average daily dose at cycle 3 was 21.1 mg/m^2^ (*n* = 6). Steady-state plasma concentrations were reached by day 15, cycle 1. No tumor responses were observed, but three patients had stabilization of the disease (50%). Median progression-free survival was 5.8 months (95% CI 2.3—not reached). There were no serious adverse events.

**Conclusions:**

The tolerable dose of sunitinib in chemotherapy-naïve pediatric patients is at least 20 mg/m^2^ on schedule 4/2. The safety profile and PK of sunitinib in pediatric patients with GIST are comparable to those in adults.

## Introduction

Gastrointestinal stromal tumor (GIST) is a tumor of mesenchymal origin occurring in the gastrointestinal tract. Most GISTs are diagnosed in adults with median age in the mid-60 s [1]. GIST arises most often in the stomach (60% of patients) or the jejunum/ileum (30%), and less commonly in the duodenum, colon, rectum, appendix, and esophagus (< 1–5%) [2]. GIST is very rare in pediatric patients, accounting for just 1–2% of all GIST cases, and has a different clinical behavior and biology compared with typical adult GIST [2–4]. Unlike adult sporadic GIST, pediatric GIST often affects females (74%) and has a higher proportion of tumors located in the stomach (85%) [2–4]. Another distinct feature of pediatric GIST is the presence of multifocality, which was reported in 23% of the patients [2, 4]. Metastases to lymph nodes, peritoneum, and liver are common in pediatric GIST [2, 4]. GIST in pediatrics is frequently an indolent disease, although 5-year progression-free survival (PFS) of 63% for a cohort of German/Austrian patients with localized or metastatic GIST has been reported [5].

Pediatric GIST rarely has genetic mutations in *KIT* or platelet-derived growth factor receptor alpha (*PDGFRA*), which are commonly present in adult GIST [6–8]. GIST in children and young adults is often characterized with mutations in the genes encoding the subunits of the succinate dehydrogenase (SDH) enzyme complex [9–11]. Another feature in pediatric wild-type GIST is a higher expression of insulin-like growth factor 1 receptor (IGF1R) compared with adult wild-type GIST [7, 12].

Testing for mutations in *KIT* and *PDGFRA* before therapy initiation is strongly recommended [13, 14]. If no mutation is identified, immunohistochemistry for SDHB is recommended, particularly for GIST occurring in pediatric patients [13, 15]. Surgery remains the standard treatment for localized GIST and, depending on risk classification and type of mutations, adjuvant treatment with imatinib may be recommended [13, 15]. Imatinib remains the standard treatment for patients with advanced unresectable or metastatic non-SDH-mutated GIST [13].

For adult patients with confirmed intolerance to or progression on imatinib, the standard second-line treatment is sunitinib [13, 15]. Sunitinib was approved by both the US Food and Drug Administration and the European Medicines Agency for the treatment of GIST after disease progression on or intolerance to imatinib [16]. This decision was based on a phase III trial showing a significant improvement in time to tumor progression with sunitinib vs placebo in imatinib-resistant/intolerant patients with advanced GIST (hazard ratio 0.33; *P* < 0.0001; median 6.3 vs 1.5 months, respectively) [17]. Sunitinib was administered at 50 mg (i.e., equivalent to approximately 30 mg/m^2^) once daily on a 4-weeks-on/2-weeks-off schedule (schedule 4/2) in this study.

There is currently no consensus among pediatric oncologists on which drug to use in metastatic or recurrent wild-type pediatric GIST. There may be a biological and clinical rationale to use sunitinib as first-line treatment [7, 18].

A phase I study, which evaluated the safety and tolerability of once-daily sunitinib administered on schedule 4/2 in patients aged 2–21 years with relapsed or refractory solid tumors, established 15 mg/m^2^ per day as the maximum tolerated dose (MTD) for patients without prior cardiac radiation or anthracycline exposure [19] and is lower than the recommended phase 2 dose in adults (50 mg). The pharmacokinetic (PK) analysis at this MTD showed lower plasma drug concentrations compared with those observed in adult patients with GIST treated with 50 mg on a schedule 4/2. However, since most of the patients treated on the pediatric phase 1 trial were heavily pre-treated with chemotherapy, it can be anticipated that in chemotherapy-naïve pediatric patients (such as patients with GIST), the tolerated dose may be higher.

We report results from a single-arm, multicenter, multinational, phase I/II clinical trial that evaluated the PK, safety, and preliminary antitumor efficacy of sunitinib in pediatric patients diagnosed with advanced, unresectable GIST (Eudra CT 2011-002008-33; ClinicalTrials.gov NCT01396148). Since the study permitted an intra-patient dose escalation based on individual patient safety/tolerance and real-time PK (cycle 1 samples), it also investigated whether doses greater than the established MTD were tolerated in these patients.

## Patients and methods

### Patient population

Eligible patients were aged 6 to < 18 years with advanced, unresectable GIST for which there were no available options for treatment with curative intent. Patients had to have non-mutant *KIT* or *PDGFRA GIST*, or demonstrated either disease progression on or intolerance to imatinib mesylate. Measurable or evaluable disease was required as per Response Evaluation Criterion in Solid Tumors (RECIST) version 1.1 and patients had to have adequate renal, hepatic and bone marrow function. Patients aged ≥ 11 years had to have Eastern Cooperative Oncology Group Performance Status 0–2, and patients aged < 11 years had to have Lansky score ≥ 50%. Patients had to have tumor tissue available to assess *KIT, PDGFRA*, and *BRAF* genotypes, and SDHB protein expression by immunohistochemistry.

Patients were excluded if they were receiving treatment with another investigational agent and/or systemic anticancer therapy within 4 weeks before sunitinib treatment initiation, or had received prior sunitinib treatment or therapy with known risk for cardiovascular complications.

### Study design and treatment

The primary objective of this phase I/II clinical trial was to characterize the plasma PK profile of sunitinib in pediatric patients with advanced, unresectable wild-type GIST. Secondary objectives were to investigate whether doses greater than the previously established pediatric MTD were tolerated in pediatric patients with GIST, evaluate safety and tolerability, antitumor activity, and explore PK/pharmacodynamic (PD) relationships with respect to safety and efficacy in these pediatric patients.

This study was conducted in compliance with the Declaration of Helsinki and the International Conference on Harmonisation Good Clinical Practice Guidelines. The final protocol, any amendments, and informed consent were approved by the institutional review board or independent ethics committee at each study center or country. All patients and/or their parent/legal guardian signed the informed consent before inclusion of the patient.

The study aimed to enroll 15 evaluable patients with GIST aged 6 to < 18 years. A protocol amendment was implemented to reduce the number of enrolled patients (aged 6 to < 18 years) from 15 to 6 evaluable patients because of the rarity of the disease and slow enrollment, and the fact that this number of patients allowed the analysis of the primary endpoints, i.e., the characterization of the PK profile in pediatric patients. The starting dose of sunitinib was 15 mg/m^2^ per day administered orally on a schedule 4/2, and patients could continue for up to 18 cycles over a treatment period of 24 months.

Patients were monitored for toxicity, with the sunitinib dose adjusted according to individual patient tolerance at the investigator’s discretion. Intra-patient dose escalation of sunitinib was allowed after completion of cycle 1 and/or later cycles, based on individual patient tolerability, and supported by real-time PK (cycle 1 samples).

Intra-patient dose escalation was in increments of 7.5 mg/m^2^ up to a maximum dose of 30 mg/m^2^ (not to exceed 50 mg/day), and dose reduction was in decrements of 7.5 mg/m^2^. In case of grade 3 toxicities, sunitinib was held until the toxicity decreased to ≤ grade 1 or ≤ 2 grade for hematologic toxicities, at which point sunitinib was resumed at the same dose level or reduced by 1 level (investigator’s discretion). In case of grade 4 hematologic toxicities the dose was reduced by 1 level after decrease to ≤ 2 grade. Re-escalation was permitted with appropriate supportive care and monitoring at the discretion of the investigator. Specific guidelines for hypertension management were provided. Any patient requiring > 4 weeks of dose interruption for toxicity was considered for study withdrawal.

### Pharmacokinetics

Blood samples for PK analysis of sunitinib and its active metabolite (SU012662) were obtained at 2, 4, 6, and 8 h post-dose on day 1, cycle 1. Trough/pre-dose samples were collected on days 1, 15, and 28 of cycle 1 and on days 1 and 28 of cycles 2 and 3. In addition, trough PK sample collection on day 15 of cycles 2 and 3 was required only if the patient underwent dose escalation during that cycle.

Standard plasma PK parameters—including maximum plasma concentration (*C*_max_), trough plasma concentration (*C*_trough_), time to *C*_max_ (*T*_max_), and area under the curve for concentration vs time profile from time 0–8 h post-dose (AUC_8_) for sunitinib and SU012662—were estimated using non-compartmental analysis (NCA) methods. Nominal sample collection times were used for NCA of sunitinib and SU012662. During the study, PK data from cycle 1 in each patient were compared with the historical data in adult patients and passed to the investigator to support and facilitate the dose-escalation process in individual patients.

### Efficacy

Magnetic resonance imaging or computed tomography scans were used for tumor measurements at screening and after every even cycle, and a fluoro-deoxyglucose positron emission tomography (FDG-PET) scan to assess tumor metabolic activity (at day 28 of cycle 1). A repeated FDG-PET scan was optional to the investigator’s discretion. Antitumor efficacy was determined based on the investigator’s objective tumor assessments according to RECIST v1.1. In case of objective tumor response (partial or complete response), confirmatory imaging studies were performed at least 4 weeks after initial documentation of response. Designation of best response of stable disease required the criteria to be met at least once after the first dose of sunitinib, at a minimum interval of 8 weeks. In case of clinical benefit, patients were allowed to continue on study drug even if progressive disease was noted.

### Safety

Safety was assessed by type, incidence, severity (graded by the National Cancer Institute Common Terminology Criteria for Adverse Events, version 4.0), timing, seriousness, and relatedness of adverse events (AEs) and laboratory abnormalities. All AEs (serious and non-serious) occurring on or after the first day of study treatment and ≤ 28 days after the last dose were considered as treatment-emergent AEs. *Medical Dictionary for Regulatory Activities* (MedDRA, version 20.0) coding was applied. Bone age imaging, echocardiography and electrocardiogram (ECG) were performed at screening.

### Statistical analysis

The efficacy analysis was based on the full analysis population, which included all enrolled patients regardless of what treatment, if any, they received. The safety analysis was based on the as-treated population, which included all enrolled patients who received at least one dose of study drug. The PK analysis was based on the PK population, which included all treated patients with at least one PK observation.

Descriptive statistics for observed and dose-corrected (where appropriate) PK data were reported for all patients with at least one PK observation. The dose correction for PK parameters was performed to account for any intra-patient dose changes from the initial dose of 15 mg/m^2^. The correction factor initial dose/current dose was multiplied by exposure parameters to obtain the dose-corrected PK parameters. Additionally, geometric mean and 95% confidence interval (CI) for the geometric mean were reported where appropriate. Summary descriptive statistics and listings of plasma concentrations by nominal time and day, and PK parameters were presented for sunitinib, SU012662, and the sum of both molecules (total drug). All concentrations below the limits of quantitation were set to zero.

In patients with day 1 PK profile-sampling data, the terminal elimination phase could not be adequately characterized to calculate the elimination half-life; therefore, the area under the plasma concentration–time curve (AUC) from time 0 to infinity, and subsequently the apparent oral clearance or steady-state total plasma exposure (AUC from time 0–24 h post-dose) for sunitinib and its main active metabolite (SU012662) could not be reliably estimated. However, these parameters were estimated using nonlinear mixed effects modeling (NONMEM) approaches and reported separately as part of an integrated population modeling analysis report.

Efficacy endpoints included objective response rate (ORR), duration of response, PFS, and overall survival. When possible, time-to-event endpoints were summarized using Kaplan–Meier methods. Safety data were summarized descriptively.

For the PK/PD analyses, the PK-evaluable patients on day 28, cycle 1 were divided into two subgroups: those with total drug trough plasma concentration (*C*_trough_) values < median *C*_trough_ value (lower exposure), and those with total drug *C*_trough_ values ≥ median *C*_trough_ value (higher exposure). The incidence of AEs (nausea, vomiting, diarrhea, fatigue, palmar–plantar erythrodysesthesia, neutropenia, thrombocytopenia, lymphopenia, anemia, and hypertension) occurring during cycles 1–3 was summarized by grade for both PK subgroups. Pearson correlation coefficients (*R*) between the percentage change in the laboratory values for absolute neutrophil count, platelet count and lymphocyte count with total drug C_trough_ values were also calculated with respect to PK visits on day 28 of cycles 2 and 3. The laboratory value nearest to the time of PK sample collection was used for correlation purposes. In addition, ORR, stable disease, and PFS were summarized for the two PK subgroups.

## Results

### Patients

Eight patients were screened, six of whom were enrolled and included in the PK, safety, and efficacy analyses. Two patients were screened but not enrolled because of the lack of measurable disease (*n* = 1) and patient/parents opting for extensive surgery (*n* = 1).

Patient demographics and baseline characteristics are presented in Table 1. Of the six enrolled patients, none had prior radiation therapy. Five patients had undergone at least one previous anticancer surgery and one patient had a biopsy of a gastric and a liver lesion before enrollment. For the 5 patients with prior surgery, the interval to enrollment was 4, 13, 16, 20, and 82 months. Two patients had received previous systemic anticancer treatments: one received adjuvant imatinib (400 mg daily) for 16 months, and the other received neoadjuvant imatinib (200 mg daily) for 11 months. Four patients received sunitinib as first-line treatment, being enrolled in this study. All but one patient had reached puberty with 3 out of 6 having open growth plates on wrist X-ray.

**Table 1:** Patient demographics and baseline characteristics (*N* = 6)

Characteristics	
Sex	
Male	1
Female	5
Age, years	
Median (range)	14.0 (13.0–16.0)
Race	
White	5
Asian	1
ECOG PS 0	6
Number of involved disease sites	
1	2
2	1
3	3
Involved disease sites	
Liver	4
Lung	1
Peritoneum	3
Stomach	3
Other*	2

*ECOG PS* Eastern Cooperative Oncology Group Performance Status, *SD* standard deviation

*Region of celiac axis and adrenal gland mass (1 each)

In all six patients, there were no detectable alterations in *KIT* and *PDGFRA*. In two patients for whom data were available, there were no detectable alterations in *BRAF*. The expression of SDHB was negative in five patients, and not tested in one patient. Methylation of *SDHC* promotor was detected in 1 patient.

### Treatment and safety

Median treatment duration was 7.2 months (range, 3.6–24.4). In all six patients treated, mean ± standard deviation (SD) cumulative dose was 4866.67 ± 2350.34 mg for the cycles administered, with a mean relative intensity of 97.62 ± 3.99%. Average daily doses of 13.5 ± 1.1, 21.1 ± 4.7, and 23.3 ± 8.4 mg/m^2^ were observed at cycles 1, 3, and 5, respectively.

Five patients discontinued treatment: four because of objective disease progression after three, four, six, and seven cycles, respectively, and one because of an AE (long lasting grade 2 anemia possibly attributed to sunitinib). Of the five patients who discontinued treatment, four were followed up for survival and completed the study phase. One patient discontinued treatment and chose not to participate in the follow-up phase.

Dose was increased in five patients to 22.5 mg/m^2^, and two patients had a further dose increase to 30 mg/m^2^ at cycles 3 and 4, respectively. Of the five patients who had a dose increase, one required a later dose reduction from 22.5 to 15 mg/m^2^ due to grade 4 neutropenia during cycle 3. A second patient, on 30 mg/m^2^, permanently discontinued treatment after five cycles due to long lasting grade 2 anemia (per protocol). Other AEs reported for this patient were grade 1 leukopenia and thrombocytopenia; both occurred during cycle 1 and were considered related to the study drug. Four patients had temporary treatment discontinuations due to treatment-emergent AEs: grade 3 neutropenia, hypoglycemia, thrombocytopenia, and grade 2 neutropenia (each *n* = 1).

In total, 82 treatment-emergent AEs were reported in six patients. Grade 4 AEs were reported in 2 patients: grade 4 subcapsular hepatic hematoma and intra-abdominal hemorrhage (*n* = 1), both of which were determined by the investigator to be related to disease progression based on laparotomy showing multiple lesions localized at stomach wall, liver, lymph node at falx hepatis, and massive peritoneal dissemination, with hemorrhagic ascites; and grade 4 neutropenia (*n* = 1). Grade 3 AEs were reported in 4 patients: neutropenia, thrombocytopenia, hypoglycemia, and hypophosphatemia (each *n* = 1). There were no deaths due to toxicity or serious AEs reported in this study. Most commonly occurring AEs included headache (*n* = 4, grade 1 or 2), diarrhea (*n* = 3, grade 1 or 2), nausea (*n* = 3, grade 1), neutropenia (*n* = 3, grades 2–4), or white blood cell count decreased (*n* = 3, grade 2). Only 1 grade 1 ECG alteration was observed. Repeated bone age imaging was not conclusive in terms of growth-plate fusion impairment. Treatment-emergent/all-causality AEs and laboratory abnormalities are shown in Table 2.

**Table 2:** Treatment-emergent, all-causality adverse event and laboratory abnormalities occurring in at least two patients (*N* = 6)

	Grade 1	Grade 2	Grade 3	Grade 4	Total
Adverse event, *n*					
Blood and lymphatic system disorders					
Anemia	1	1	0	0	2
Neutropenia	0	1	1	1	3
Thrombocytopenia	0	1	1	0	2
Nervous system disorders					
Headache	3	1	0	0	4
Gastrointestinal disorders					
Diarrhea	2	1	0	0	3
Nausea	3	0	0	0	3
Dyspepsia	2	0	0	0	2
Investigations					
White blood cell count decreased	0	3	0	0	3
Metabolism and nutrition disorders					
Decreased appetite	1	1	0	0	2
Musculoskeletal and connective tissue disorders					
Back pain	2	0	0	0	2
Laboratory abnormalities, *n*					
Chemistry					
Creatinine	5	1	0	0	6
Hyperglycemia	4	1	0	0	5
Aspartate aminotransferase	3	0	0	0	3
Alanine aminotransferase	2	0	0	0	2
Amylase	2	0	0	0	2
Hypermagnesemia	2	0	0	0	2
Hypocalcemia	2	0	0	0	2
Hypoglycemia	0	1	1	0	2
Hypophosphatemia	1	0	1	0	2
Hematology					
Lymphopenia	6	0	0	0	6
Neutrophils (absolute)*	0	4	1	1	6
White blood cells*	0	6	0	0	6
Platelets*	3	0	1	0	5
Anemia	2	1	1	0	4

Maximum CTCAE grade is defined as the maximum CTCAE grade value for the specific ‘Preferred Term’ or laboratory parameter. CTCAE version 4.0 was used. MedDRA (version 20.0) coding dictionary applied

*CTCAE* common terminology criteria for adverse events, *MedDRA* medical dictionary for regulatory activities

*Values are for patients with a decrease in the blood cell count

### Pharmacokinetics

At an oral dose of 15 mg/m^2^ in pediatric patients with GIST, median *C*_max_ for sunitinib and SU012662, respectively, was 18.4 and 2.37 ng/mL; median *T*_max_ was 8 h for both (Table 3). AUC_8_ was 82.7 and 10.7 ng.h/mL for sunitinib and SU012662, respectively. The observed and dose-corrected *C*_trough_ on day 15, cycle 1, and on day 28, cycles 1, 2, and 3 are shown in Table 3. The coefficient of variation (%) in steady-state observed or dose-corrected *C*_trough_ on day 28, cycle 1 was 46%, 36%, and 42% for sunitinib, SU012622, and total drug, respectively (Table 3).

**Table 3:** Summary of pharmacokinetic parameters following sunitinib oral doses (starting dose of 15 mg/m^2^) in pediatric patients with GIST

PK parameter	Sunitinib	SU012662	Total drug
Observed (*N* = 6)			
*T*_max_, h	8.0 (4.0–8.0)	8.0 (4.0–8.0)	NC
*C*_max_, ng/mL	18.4 (34) [16.1]	2.37 (17) [2.44]	NC
AUC_8_, ng⋅h/mL	82.7 (39) [80.0]	10.7 (35) [9.82]	NC
*C*_trough_ C1D15, ng/mL	24.4 (42) [20.8]	11.7 (15) [11.7]	36.0 (31) [32.4]
*C*_trough_ C1D28, ng/mL	29.1 (46) [29.3]	13.0 (36) [12.8]	42.1 (42) [42.1]
*C*_trough_ C2D28, ng/mL	44.7 (90) [30.9]	20.9 (63) [15.9]	65.6 (80) [48.7]
*C*_trough_ C3D28, ng/mL	31.3 (49) [27.8]	20.5 (46) [19.5]	51.8 (46) [43.5]
Dose-corrected (*N* = 6)			
*C*_trough_ C1D15, ng/mL	24.4 (42) [20.8]	11.7 (15) [11.7]	36.0 (31) [32.4]
*C*_trough_ C1D28, ng/mL	29.1 (46) [29.3]	13.0 (36) [12.8]	42.1 (42) [42.1]
*C*_trough_ C2D28, ng/mL	32.5 (69) [24.9]	15.2 (45) [14.8]	47.7 (61) [38.9]
*C*_trough_ C3D28, ng/mL	19.9 (36) [18.6]	13.1 (31) [13.8]	32.9 (31) [29.8]

Values are mean (CV%) [median] for all, except median (range) for *T*_max_

*AUC*_8_ area under the curve for concentration vs time profile from time 0–8 h post-dose, *C* cycle, *C*_*max*_ maximum plasma concentration, *C*_*trough*_ trough plasma concentration, *CV%* coefficient of variation, *D* day, *GIST* gastrointestinal stromal tumor, *NC* not calculated, *T*_*max*_ time to first occurrence of maximum observed plasma concentration

### Pharmacokinetics/pharmacodynamics

The relationship between incidence of selected all-grade AEs and total drug plasma concentrations is shown in Table 4. The Pearson correlation coefficient (*R*) for the relationship between percentage change in selected safety laboratory values and total drug *C*_trough_ on day 28 of cycles 2 and 3, respectively, was − 0.59 and − 0.55 for absolute neutrophil count, and − 0.64 and − 0.66 for platelet count, indicating an overall moderate negative correlation (− 0.7 < *R* ≤ − 0.5). The *R* value for the relationship between percentage change in lymphocyte count and total drug *C*_trough_ on day 28 of cycles 2 and 3, respectively, was − 0.48 and − 0.29, indicating an overall weak negative correlation (− 0.5 < *R* ≤ − 0.3).

**Table 4:** Relationship between incidence of selected adverse events and plasma drug exposures

Adverse event	All evaluable patients	
	< Median total drug* (*n* = 3)	≥ Median total drug* (*n* = 3)
Nausea	0	2 (grade 1)
Vomiting	0	1 (grade 1)
Diarrhea	0	2 (grades 1 and 2)
Fatigue	0	1 (grade 2)
Palmar–plantar erythrodysesthesia syndrome	1 (grade 1)	0
Neutropenia	2 (grades 2 and 4)	1 (grade 3)
Thrombocytopenia	1 (grade 1)	1 (grade 2)
Lymphopenia	0	0
Hypertension	0	0
Anemia	1 (grade 1)	0

Total Drug Concentration (ng/mL), sunitinib plus SU012662 drug concentration (ng/mL)

*Total drug trough plasma concentration (*C*_trough_) values < median *C*_trough_ value (lower exposure), and total drug *C*_trough_ values ≥ median *C*_trough_ value (higher exposure)

The rate of stable disease was 33.3% in the PK subgroup with total drug *C*_trough_ < median and 66.7% in the PK subgroup with total drug *C*_*t*rough_ ≥ median. Median PFS was 2.6 months in the lower exposure PK subgroup and 9.0 months in the higher exposure PK subgroup on day 28, cycle 1. The *R* for the relationship between PFS and total drug *C*_trough_ on day 28, cycle 1 was 0.59, indicating a moderate positive correlation (0.5 ≤ *R* < 0.7).

### Efficacy

After two cycles of sunitinib, three patients had stable disease and three had disease progression as assessed by CT or MRI. The three patients with disease progression continued on study medication because the investigators deemed there was clinical benefit to continue study medication. One of the patients had a modest increase in sum of diameters (+ 23%) while having had a low drug exposure during cycle 1, due to dose rounding (13.3 mg/m^2^) and probable drug interactions with grapefruit juice. This patient had a progressive increase in dose up to 31 mg/m^2^, but went off study after six cycles because of further disease progression. The second patient had a 46% increase in diameter at cycle 2 but remained on study because of clinical benefit and had an increased dose to 21.7 mg/m^2^. The patient went off study after three cycles because of the appearance of new lesions. The third patient had a 50% increase in the target lesion at the evaluation after cycle 2. The dose was increased to 19.7 mg/m^2^ at cycle 3 but had to be decreased at cycle 4 because of AE (neutropenia). The patient went off study after 4 cycles because of further disease progression.

Serial FDG-PET imaging was available for 4 of the 6 patients but did not reveal any significant decrease in metabolic activity with sunitinib treatment.

Of the six patients, three had stable disease and three had disease progression as best response. No complete or partial responses were observed. One patient remained on study for the 18 cycles allowed and had stable disease. The patient who discontinued treatment due to a grade 2 anemia had stable disease at the time of treatment withdrawal. Median PFS in the four patients was 5.8 months (95% CI 2.3–not reached). The time from first study dose to last available survival follow-up as per protocol ranged from 0.9 to 2.4 years for the six patients.

## Discussion

Despite fewer pediatric patients being recruited than originally planned in this study, we were able to characterize the PK profile of sunitinib, which was the study’s primary endpoint. At a daily oral dose of 15 mg/m^2^ in pediatric patients with GIST, both sunitinib and SU012662 reached steady-state plasma concentrations by day 15, cycle 1, with no additional accumulation across cycles. The dose-corrected day 28, cycle 2 steady-state mean *C*_trough_ in this study was comparable to the previously reported steady-state *C*_trough_ on day 14, cycle 1 in pediatric patients with refractory solid tumors, respectively, for sunitinib (32.5 vs 33.0 ng/mL), SU012662 (15.2 vs 16.0 ng/mL), and total drug (47.7 vs 49.0 ng/mL) [19]. However, the dose-corrected (i.e., to 15 mg/m^2^) *C*_trough_ for sunitinib, SU012662, and total drug in pediatric patients with GIST were lower than those in adult patients with GIST (32.5 vs 42.6, 15.2 vs 18.9, and 47.7 vs 62.2 ng/mL, respectively) at the dose of 50 mg once daily on schedule 4/2 (Data on file). Therefore, pediatric doses higher than 15 mg/m^2^ would be required to achieve the plasma drug exposures comparable to those achieved in adults with GIST at the dose of 50 mg once daily on schedule 4/2.

Considering that the majority of pediatric patients had their initial dose escalated to approximately 22.5 mg/m^2^ during cycle 2, the observed *C*_trough_ values during cycle 2 are in fact higher than the dose-corrected *C*_trough_ values actually reflecting the dose escalation to 22.5 ng/m^2^ dose. Furthermore, the proportional calculated *C*_trough_ values on day 28, cycle 2 at a dose of 20 mg/m^2^ in pediatric patients with GIST are 43.3, 20.3, and 63.6 ng/mL for sunitinib, SU012662, and total drug, respectively, and comparable to those observed in adult patients with GIST at 50 mg once daily on schedule 4/2. Therefore, considering that steady-state total plasma exposures (i.e., AUC) are highly correlated with steady trough plasma concentrations for sunitinib and SU012662, a starting dose of 20 mg/m^2^ in pediatric patients with GIST would be expected to provide drug exposures comparable to those in adult patients with GIST at the dose of 50 mg on schedule 4/2.

The best overall response in this study was stable disease in 50% of patients (*n* = 3) and for three patients with PD there seemed to be a clinical benefit justifying a continuation of study medication for additional cycles. This clinical benefit was based on either moderate metabolic response on FDG-TEP scan or a mixed response observed on CT-scan or MRI. A previous study by Janeway et al. also demonstrated antitumor activity with sunitinib in imatinib-resistant pediatric patients with GIST [20]. Of the seven sunitinib-treated pediatric patients with GIST, one had partial response, five had stable disease, and one had disease progression [20]. The duration of partial response/stable disease was 7 to > 21 months, with an average of 15 months. Two patients had sunitinib for more than 18 and 21 months for sustained partial response/stable disease. In five of the six patients with partial response/stable disease, sunitinib resulted in a longer time-to-progression (TTP) than that achieved during imatinib treatment. The difference in TTP on sunitinib vs prior imatinib ranged from 2 to 17 months, with an average of 7.5 months [20].

In the series reported by Rutkowski et al., a best response of stable disease was observed in seven of the nine patients treated with sunitinib, and all but one patient eventually had disease progression [21]. Among the eight patients who progressed, PFS and TTP duration ranged from 1 to 28 months, while one patient remained progression free after 73 months (as per date of data cut-off) [21]. In all previous publications [7, 20, 21], stabilization of the disease has been observed in the majority of pediatric patients, with 13/20 patients with stable disease and 2/20 patients with partial response. As for treatment duration, 14/20 patients had ≥ 6 months of sunitinib, while 10/20 patients had ≥ 12 months of sunitinib treatment, and two patients > 2 years. This observation is suggestive of antitumor activity for a substantial proportion of pediatric patients with advanced GIST [7, 20, 21].

The correlation analysis between PK and efficacy seemed to show a higher rate of stable disease in patients with higher total drug *C*_trough_ on day 28, cycle 1, although no statistical analysis was performed due to small patient numbers in each subgroup (i.e., *n* = 3). Median PFS was also longer in patients with higher total drug *C*_trough_. The moderate positive correlation between PFS and total drug *C*_trough_ demonstrates that sunitinib can be more effective at higher drug plasma concentrations in pediatric patients with GIST. These results are consistent with a previous study showing that increased exposure to sunitinib was associated with improved antitumor activity in patients with GIST or metastatic renal cell carcinoma [22].

There were no unexpected safety findings with sunitinib in this study population of pediatric patients with GIST. Doses higher than the previously defined maximum tolerated dose (15 mg/m^2^/day) [19] were generally well tolerated in this limited population (increased to 22.5 mg/m^2^/day in five of six patients and further increased to 30 mg/m^2^/day in two patients). The safety profile was consistent with previous reports of sunitinib safety in adult patients with solid tumors [17, 23] and the well-defined toxicity profile for sunitinib in adult patients with GIST. The AEs were manageable with standard medical intervention and/or dose modification in this population. Moreover, our results are in line with three other studies [7, 20, 21] in pediatric patients with GIST showing that sunitinib treatment is generally well tolerated at dose levels that were higher than the previously reported MTD of 15 mg/m^2^/day [19]. The correlation analysis showed an overall moderate negative correlation between percentage change in absolute neutrophil or platelet count and total drug *C*_trough_. This indicates a higher degree of on-target modulation at higher plasma drug concentrations in pediatric patients with GIST.

In conclusion, this study of six pediatric patients with GIST found that both sunitinib and SU012662 reached steady state by day 15, cycle 1, with no additional accumulation across cycles. Higher total drug plasma concentrations were potentially associated with a higher likelihood of stable disease and on-target AEs (e.g., hematological), although not statistically significant. AEs were in general tolerable and clinically manageable. A starting sunitinib dose of 20 mg/m^2^ in pediatric patients with GIST would be expected to provide drug exposures comparable to those in adult patients with GIST at the dose of 50 mg on schedule 4/2.
